# The Nasal Tip Rotation After Primary Rhinoplasty Using Columellar Strut Graft

**DOI:** 10.7759/cureus.14152

**Published:** 2021-03-28

**Authors:** Yazeed Alghonaim, Fahad Alobaid, Jury Alnwaiser

**Affiliations:** 1 Otolaryngology, King Saud Bin Abdulaziz University for Health Sciences, Riyadh, SAU; 2 Otorhinolaryngology, King Abdulaziz Medical City, Riyadh, SAU

**Keywords:** nasolabial angle, rhinoplasty, tip rotation

## Abstract

Objective: In this study, we aimed to investigate the degree of nasal tip rotation three months after rhinoplasty using columellar strut graft.

Methods: Using photographs of 25 patients who underwent rhinoplasty, we prospectively analyzed nasal tip rotation before, during, and after three months of the surgery. Columellar strut graft was used for all patients for tip support. Standardized left profile images were taken. For evaluation of tip rotation, the nasolabial angle was measured. The results were statistically compared, and a p value less than 0.05 was considered statistically significant.

Results: The mean preoperative nasolabial angle (NLA) for the entire group was 91.44°, and the directly postoperative angle measured 108.84°. The mean of postoperative nasolabial angles measured at three-month follow-up was 97.2°. The preoperative, directly postoperative, and three-month postoperative nasolabial angles were all recorded.

Conclusion: Considering the results of this study, a postoperative nasolabial angle is increased compared to preoperative angle. However, an occasional dropping of the angle might be seen in following months, which can be linked to several factors.

## Introduction

In the medicine field, constant inventions and improvements are being made throughout the years to improve the former methods that were previously applied. To be more specific, reconstructive and cosmetic surgeries are among the surgical divisions that have undergone a significant transformation over the years. The techniques involved in reconstructive head and neck surgery are aimed to improve an individual’s appearance cosmetically or to repair some forms of congenital anomalies, such as the impaired placement of the nasal septum.

There are various types of nasal surgeries. Rhinoplasty is among the most typical nasal surgeries, which involves elevation of the nasal tip to make the nose appear more prominent [1]. The effect of nasal tip rotation is directly proportional to the overall impact of the reconstructive surgery in creating a redefined appearance of other facial structures such as the lips and the nasolabial folds [2]. Increasing the angle between the nose and the labial folds results in increased length of the lower third of the face [2]. The result of the surgery is that there would be an increased length between the nasal tip and the upper lip, with a flattened nasolabial ridge. In some patients, the upper lip length was noted to decrease progressively with time, although not up to the size it was before the operation [2].

In Saudi Arabia, rhinoplasty is among the top five most frequently done cosmetic surgeries [3]. Moreover, a study conducted in Saudi Arabia concluded that more than 90% of patients interested in rhinoplasty aim for smaller nose size and narrower nostrils [3]. Almost 60% of these patients asked for their nasal tip to be pointing up, and 82% wanted it to be sharp [3]. Another study including 62 patients who underwent rhinoplasty using columellar struts technique found that nasal tip projection increased from 0.58 to 0.60 postoperatively [4]. Additionally, nasolabial angle (NLA) increased from 93.96° to 100.92° [4]. Consequently, the result of the nasal tip immediately post-surgery is usually satisfying as it achieves all the concerns of the patients [4]. However, these results usually change gradually. Four to six weeks after rhinoplasty, we observe occasional dropping of the nasal tip due to several causes [5]. Such causes may include scar contractures, weight of the nose, and the action of the depressor septi nasi muscle [5]. In our study, we will focus on time as an independent variable and its effect on the degree of the nasal tip rotation and the NLA.

## Materials and methods

**Figure 1:**
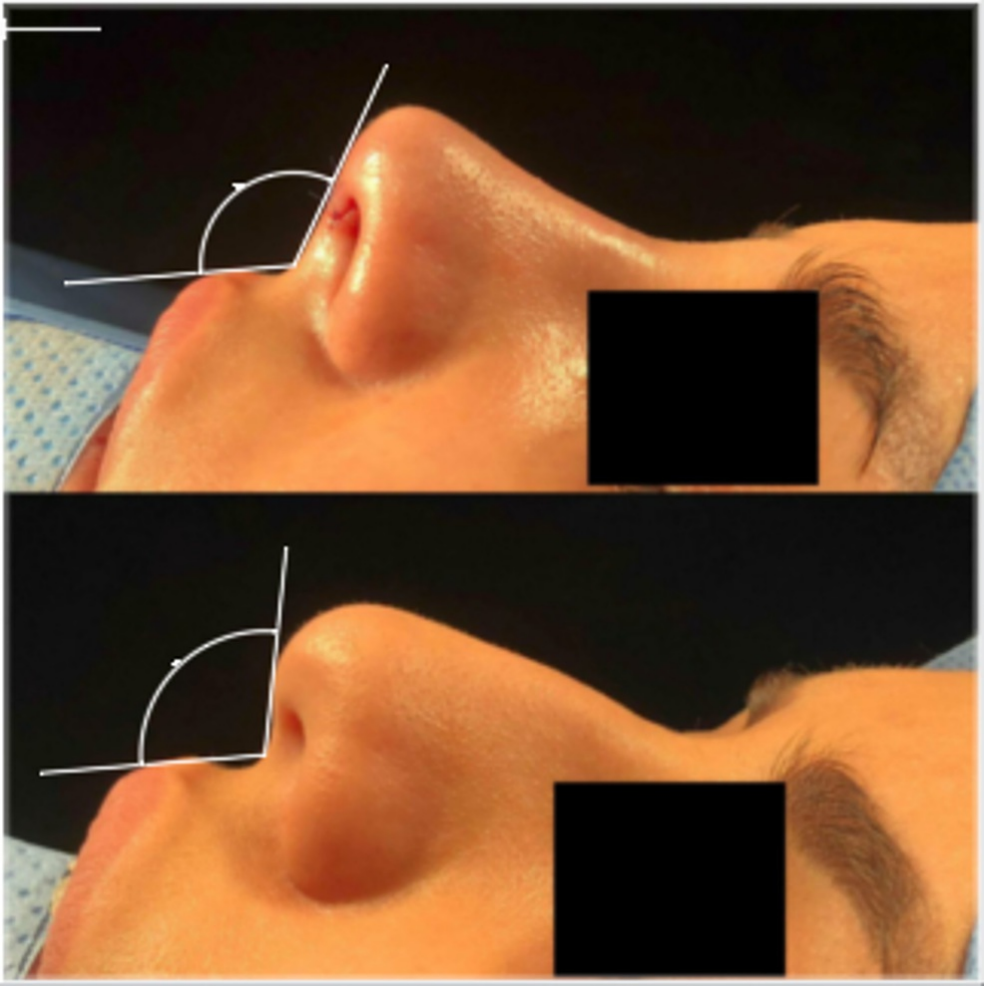
Nasolabial angle measured postoperatively (in the operation room)

**Figure 2:**
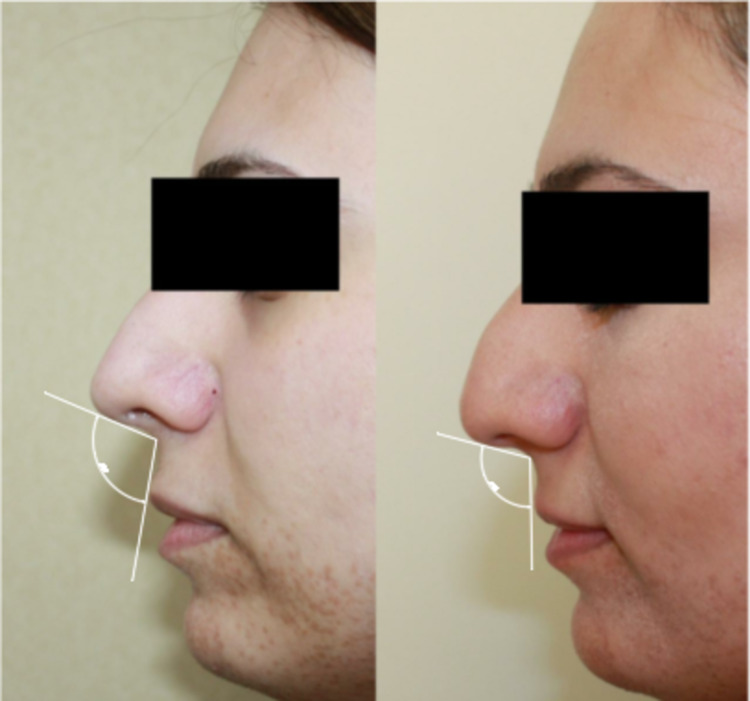
Comparison of the nasolabial angle preoperatively and three months postoperatively

Twenty-five patients who underwent primary rhinoplasty with columellar strut graft were included in our study. These patients were operated by the same surgeon at the same center. Patients who had any other modality as primary intervention for tip rotation or had revision rhinoplasty were excluded. Columellar strut graft was the only graft used in our patients. We collected the data prospectively. Standardized lateral left sided images were taken for the patients preoperatively (t1), directly postoperatively (t2), and three months postoperatively (t3). Using Canon digital camera, we took profile view pictures with a plane background at a fixed distance of 70 cm. We used two external light sources to keep photographs shadowless. Also, we aligned the Frankfort line within the grid of the camera. The analysis was blinded and done by two other observers. For the evaluation of nasal tip rotation, the NLA was measured using a computerized program called AutoCAD (Autodesk, CA, USA), and the results were recorded. Two lines were drawn parallel to the upper lip and columella to measure the NLA (Figures 1, 2). The data were analyzed by SPSS software program version 23 (IBM Inc., Armonk, USA). Angle degrees were reported as categorical variables. The mean and standard deviation were calculated, and the difference between them was assessed using paired sample t-test. Any result with a p value less than 0.05 was considered statistically significant.

## Results

Of the 25 patients included in this study, all of them were females with an average age of 29 years old ranging from 21 to 46 years old. In both early and late postoperative periods, no major or minor complications were encountered in all 25 patients.

The mean preoperative NLA for the entire group was 91.44°, and the directly postoperative angle measured 108.84°. The mean of postoperative NLA measured at three-month follow-up was 97.2°. The preoperative, directly postoperative, and three-month postoperative NLA were all recorded (Table 1).

**Table 1:** Descriptive analysis T1, Preoperative angle; T2, day 0 postoperative angle; T3, three-month postoperative angle.

Descriptive analysis					
	N	Minimum	Maximum	Mean	SD
Age (Years)	25	21	46	28.92	5.74398
T1 (Degree)		68°	117°	91.44°	12.39314
T2 (Degree)		81°	124°	108.84°	11.27564
T3 (Degree)		75°	118°	97.20°	12.50333

The results were analyzed statistically, and the paired t-test was used to detect the significance between the groups. After comparing the preoperative and directly postoperative NLA, the difference was statistically significant with a p value of 0.000. Moreover, the three-month follow-up was compared to the directly postoperative photographs, and it was significant with a p value of 0.000 (Tables 2, 3).

**Table 2:** Paired sample test T1, Preoperative angle; T2, day 0 postoperative angle; Sig (2-tailed), two-tailed p value.

Paired Sample Test								
	Paired differences					t	df	Sig (2-tailed)
	Mean	SD	Std. Error mean	95% Confidence interval of the differences				
				Lower	Upper			
T1-T2	-17.4	8.72258	1.74452	-21.0005	-13.7995	-9.974	24	0,000

**Table 3:** Paired sample test T2, Day 0 postoperative angle; T3, three-month postoperative angle; Sig (2-tailed), two-tailed p value.

Paired Sample Test								
	Paired differences					t	df	Sig (2-tailed)
	Mean	SD	Std. Error mean	95% Confidence interval of the differences				
				Lower	Upper			
T2 - T3	11.64	8.50921	1.70184	8.12757	15.15243	6.84	24	0,000

## Discussion

An important role in rhinoplasty is a stable and strong nasal tip with a maintained long-term nasal tip rotation [6,7]. Rotation of the tip is measured by the NLA [8]. It differs in women and men [6]. In women, the ideal angle ranges from 95 to 110 degrees [8]. In men, however, it should be closer to 90 degrees [8]. There are many types of grafts and suture techniques that have been described to support and shape the nasal tip and maintain its stability [6]. Some of the most commonly used techniques especially in open rhinoplasty approach are dome-shaping suture and interdomal suture [6]. These techniques were used as standard techniques in all our cases. Other techniques such as columellar strut graft, septal extension graft, or septocolumellar suture are also utilized among surgeons [6]. A combination of these maneuvers is used by many surgeons to shape and construct the nasal tip [6].

In our study, we used columellar strut graft for all patients. Columellar strut graft is a popular and effective form of an invisible graft in rhinoplasty [7]. To acknowledge the importance of columellar strut graft, we have to understand the role of native columella. First of all, the columella must be positioned in a balanced space in relation to the adjacent alar rim and medial crura [7]. It can help provide support and balance to the adjacent structures [7]. A misshaped columella is not aesthetically pleasing. That being said, augmenting a cartilaginous strut to columella might be absolutely essential to provide the structural support and the nasal tip desired aesthetically [7]. Any mispositioning of the crura can be solved by some suturing techniques, along with proper placement of the columellar strut graft [7]. Rohrich et al. have published a classification and algorithm for the use of columellar strut graft [7]. He classified the graft into four types. The use of type 3, long and floating graft, and type 4, long and fixed graft, is recommended to increase nasal tip projection [6,8].

In this study, we aimed to investigate the degree of nasal tip rotation after primary rhinoplasty using columellar strut graft. We collected 25 patients undergoing primary rhinoplasty and measured their NLA preoperatively, directly postoperatively, and three months postoperatively. We found that the mean NLA increased from 91.44˚ preoperatively to 108.84˚ directly postoperatively. This was statistically significant with a p value of 0.000. We also found that the mean NLA decreased from 108.84˚ directly postoperatively to 97.2˚ three months postoperatively. This was also statistically significant with a p value of 0.000.

In a study that aimed to measure the changes in elasticity of the nasal tip using columellar strut graft versus tongue in Groove method over a year after rhinoplasty, they found that patients in both groups had increased NLA postoperatively [9]. In the columellar strut group, the mean NLA increased from 97.01° to 112.78° postoperatively [9]. Fernando et al. also found that the mean NLA increased from 92.7° to 105.5° one week postoperatively [10]. These results are consistent with our results, which stated an increase in the mean NLA postoperatively from 91.44° to 108.84°. However, in the first study, a three-month follow-up showed no difference in the NLA compared to the results postoperatively [9]. This differs from our findings, which indicates a decrease in the mean NLA from 108.84° to 97.2° after three months. A possible explanation for this might be the difference in the assessment tools or the population number. These results are also consistent with Pedroza et al., where they found that the mean NLA decreased six months postoperatively from 105.5° to 102.1° [10].

Secondary nasal tip dropping might not be desired aesthetically [5]. Fred et al. attributed this decrease in NLA to several causes [5]. These causes include scar contractures at the septocolumellar suture line, weight of the lobule, the pull-down action of the depressor septi nasi muscle, and finally failure of surgical techniques [5]. Failure of surgical techniques can be a preventable cause as suggested by Fred et al. To prevent secondary nasal dropping, Fred et al. divided the nasal tip into two different types and suggested a specific surgical technique for each one [5]. The first type is the oversized nose with adequate projection, and the second type is snub nose with inadequate projection [5]. Type 1 is more frequently operated on than type 2 and usually has more successful results regarding secondary nasal dropping [5]. Another study done by Pedroza et al. suggested that loss on nasal rotation six months after rhinoplasty might be due to the resolution of the swelling or weakening of the sutures as they used absorbable material [10]. In our study, we used absorbable sutures including polydioxanone suture (PDS) for the dome and vicryl rapide suture for the columellar strut. That being said, a decrease in rotation should be taken into consideration when planning the surgical techniques to achieve the wanted long-term results.

The main limitation to our study is the short follow-up period and the limited number of the patients. This limitation can be overcome with a longer follow-up period and a larger sample size, which would give more precise results.

## Conclusions

In conclusion, rhinoplasty is among the most typical nasal surgeries, which involves elevation of the nasal tip to make the nose appear more prominent. Various techniques are used to shape and support the nasal tip such as columellar strut graft that was used in this study. Considering the results of this study, a postoperative nasolabial angle is increased compared to preoperative angle. However, an occasional dropping of the angle might be seen in following months, which can be linked to several factors such as scar contracture, weight of the lobule, and the action depressor septi nasi muscle.
